# Transactions of the Society for the Promotion of Medicine and Surgery at Amsterdam

**Published:** 1842-04

**Authors:** 


### Art. XVII.

*Verhandelingen van het Genootschap ter Bevordering der Genees en Heelkunde te Amsterdam.* Eerste Deel. Eerste Stuk.—*Amsterdam*, 1841. 4to, pp. 54-93. With 4 Plates.

*Transactions of the Society for the promotion of Medicine and Surgery at Amsterdam.* Vol. I. Part I.—*Amsterdam*, 1841.

The first part of this volume contains an account of the general transactions of the Society since 1835, when the last volume was published by that Society, of which, under a new name and new rules, the present is the immediate successor. It includes abstracts of the communications made at their several meetings, and the oration delivered by the president, Tilanus, on the fiftieth anniversary of the establishment of the Society.

In the second and larger part, are contained five of the chief papers communicated by members of the Society, and of each of these we propose to give now a brief account.

I. *A remarkable Case of Tubercles of the Brain, by G. C. B. Suringar.* The patient was a scrofulous girl, eleven years old, in whom there slowly came on severe headach, with loss of power over the lower limbs, which gradually increased to complete paralysis of them, and of the upper limbs, and immobility of the eyeballs. Her head was in the later stages of the diseases thrown backwards, though she had some voluntary power over it: her sight was not much affected, and the iris moved freely, but, as both upper lids had fallen and nearly closed, she was obliged to move her head till the rays from what she wanted to see could enter the narrow aperture between the lids. She died about four months from the commencement of her illness, and on examination each of the optic thalami was found to contain a large mass of firm granular tuberculous matter. The rest of the brain was nearly healthy, and no tuberculous deposit could be found in any other organ.

After relating his case, the author proceeds to a judicious review of its characters as compared with those described by all the chief writers upon the disease; but of this, as most of the works he quotes are well known in England, we need perhaps give no account.

II. *Anatomical and Pathological Remarks on Inflammation of some internal parts of the Eye, and, in particular, on Choroiditis as the cause of Glaucoma, by J. L. C. Schroeder van der Kolk.* This paper contains some observations of high interest on both the healthy and morbid anatomy of the eye, which we shall give in the author's own words, although differing from him in several points, both as to the truth and the novelty of his views.

"The choroid," he says, "is nothing but a prolongation of the pia mater of the brain, on which are distributed externally the *vasa tortuosa,* as they are called, while on the inner surface or tunica ruyschiana, the vessels pass into the most delicate winding ramifications which appear to secrete the pigment. These vessels however by no means terminate at the boundary of the choroid; they go on, as is well known, to the iris, and a very beautiful expansion of them forms the *corona ciliaris,* or ciliary processes. These processes, which consist of elegant arched folds, are, in my opinion, very important parts of the eye: their folds fit into those of the *zonula zinnii,* which is placed around the lens, and constitutes the most anterior part of the *membrana hyaloidea,* or of the vitreous humour. This connexion however is, according to my observations, rendered much more intimate by a great number of the finest capillary vessels which pass from the fringe of the *corona ciliaris* into the zonula behind it, and from which there arise some most important distributions of vessels, which have not yet been accurately enough described. The arrangement has indeed been observed and figured by Henle and other anatomists in the eye of the immature fœtus; in the adult its existence has, by others, been doubted; but, in a most fortunate injection, I have very distinctly filled them in an adult healthy eye, and have found that from the outermost edge of this zonula zinnii, there pass extremely delicate vessels into the hyaloid membrane. (p. 39.)

"The vitreous humour is, according to my observations, surrounded by a very thin serous membrane, in which there run some extremely delicate vessels, which can only very rarely be filled with a fine injection. If this membrane be taken off the vitreous humour, (which I once succeeded in accomplishing in an extremely well-injected eye,) one sees that these very delicate vessels are distributed all over it: but without this previous and very difficult injection it is, in consequence of its extreme thinness and transparency, hardly possible to discern the membrane itself.

"The vessels in the membrane, which probably serve for the nutrition of the vitreous humour, arise from a double source. Some shorter ones, which for distinction's sake I shall call *vasa brevia membranæ hyaloideæ,* come from the outer edge of the zone of Zinn, whence they spread with numerous fine branches over the hyaloid membrane, and especially over its foremost part; others arise from the *arteria centralis retinæ,* immediately by its branching in the retina. These last are distinguished by their fineness and their straighter course from the vessels of the retina itself, and they pass to the *vasa brevia* just mentioned: there appear to be four of them, and I call them *vasa longa membranæ hyaloideæ.** The vessels of the vitreous humour are thus connected through the medium of the zone of Zinn with those of the ciliary processes and of the choroid: those of the retina, which are very numerous and much larger and more tortuous than those of the vitreous humour, terminate for the most part in the retina itself, but partly, as a preparation which I possess seems to show, unite with the fine vascular network of the zone of Zinn and of the ciliary processes. (p. 40.) ......

"Hence then it appears that the vessels of the choroid connect it with the iris, ciliary processes, zone of Zinn, retina, and vitreous humour. But from this general union there arise some still finer vessels. In the minutely injected eye already mentioned, it appears that some very delicate branches pass from the anterior edge of the zone and ciliary circle to the anterior surface of the capsule of the lens, (in a course, however, different from that which they have been described as taking in the fœtus.) The branch of the arteria centralis retinæ, which in the fœtus perforates the vitreous humour to distribute itself in the posterior portion of the capsule, completely disappears at a later period, and is, I suspect, replaced for the nutrition of that part by some very small branches from the zone; this, however, I have never been able to prove by injection. (p. 41.) ........

"Besides these vessels there appear also to be some serous ramifications, (which in a healthy eye are too fine to be artificially filled,) passing from the outer edge of the iris over the posterior surface of the cornea in the *membrana Demoursii;* at least, in a chronic inflammation of the eye they may be so dilated as to be filled with a fine colouring material, and in this state I possess an excellent preparation of them. ........

"Hence, then, one sees that the place from which most vessels ramify into the internal parts of the eye, and which is thus in the highest degree important, is constituted by the meeting of the vessels of the choroid and ciliary processes with those of the zone of Zinn and retina, from whence other minute branches distribute themselves into the capsule and the hyaloid membrane." (p. 41.)

From this general anastomosis of the vessels of the choroid, the author

* These last vessels have been injected by Mr. Dalrymple, (Tyrrell on Diseases of the Eye.)

thinks that its affections must have a more important influence than is generally supposed in inducing morbid changes in other parts of the eye. But his chief purpose is to show that the cause of glaucoma is a chronic inflammation of the choroid, and a consequent effusion of plastic lymph between it and the retina. The serous character of the membrane renders effusion of lymph the most natural result of its inflammation, and all the signs of glaucoma may be explained better by supposing this to have taken place than in any other way. The fulness of the eyeball, by the addition of serum and lymph to its contents, gives that sensation as if the eye were bursting, which exists in the early stages. The pressure of the retina from behind forwards, producing partial paralysis of it, an alteration from its due relation to the focus of vision, and a diminution of the anterior chamber, accounts for the loss of sight, a loss which, though it varies in its degree, is always more than is proportionate to the change of colour. The colour of the effused lymph, while it is yet soft and transparent, produces no evident change in the aspect of the pupil, though the loss of sight may be considerable; but when it is condensed it gives the peculiar hue of glaucoma, and that *deep* and *concave* appearance, as if it came from some distant hollowed surface, which cannot in any way be reconciled with the common notion that glaucoma depends on any change of the vitreous humour. With the effusion of lymph there probably also exists a diminished secretion of pigments, giving rise to the altered colour of the iris, and having some share in producing the glaucomatous hue. The immobility and dilatation of the pupil indicate an affection of the ciliary nerves: and its being generally increased in width (so as to be like the pupil of a ruminant), renders it probable that the dilatation depends, not on any disease of the retina, but on an affection chiefly of the long ciliary nerves which run horizontally by the two long ciliary arteries.

The morbid appearances found in glaucoma confirm the view of it which is here taken. The vitreous humour has never been seen to have the peculiar colour: wherever it has been altered at all, its colour has been yellow or gray or reddish; and these changes, in all probability, ensued subsequently to the change in the choroid. Neither can an amber hue of the lens account for the colour; for this change is by no means constant in glaucoma. A diminished secretion of pigment, which the author has observed in two cases of glaucoma, will not better account for the peculiar colour; for the tinge resulting from this defect is gray, and when it exists in an extreme degree, as in albinos, there is certainly no greenish hue. The real source of the peculiar character of the disease was indicated by two cases which the author was able to examine after death. In the eye of an old woman, affected with amaurosis and distinctly glaucomatous, he found a layer of plastic lymph between the choroid and retina, somewhat more than a line in thickness, occupying the posterior half of their circumference, and already seeming to contain some small vessels. In another eye he found a similar effusion in a more advanced stage, the glaucoma having existed six years, and the lymph being more organized.

Such is the chief evidence for the author's explanation of glaucoma.

The remainder of the paper is occupied by criticisms of the opinions which others have held on the nature of this disease, and contains, besides, some miscellaneous observations on the anatomy and diseases of the eye; among which the author denies (absurdly we think) the existence of the membrana jacobi, which he considers as nothing but a precipitate from the serous fluid which always exists between the choroid and retina. He observes, also, that that which is commonly described as an ossification of the retina, is in reality an ossification of a membrane between it and the choroid (a fact noticed by Van der Lith); and he describes and figures remarkable cases, in one of which he succeeded in injecting the vessels of the anterior part of the capsule in cataract with chronic inflammation of the capsule, and in another injected a fine vascular network in the membrane of the aqueous humour which had been similarly affected.

The long abstract we have given of this paper compels us to abbreviate what remains to be noticed of this volume.

III. *Subcutaneous Emphysema, especially of that in the neck coming on without any previous external injury, by W. H. de Vriese.* The patient was a little boy, six years old, who had been suffering for some time from hooping-cough, and in whom there suddenly supervened excessive dyspnœa with pain in the right side. When the author first saw the child he was in a state approaching to suffocation; but his attention was chiefly attracted by a swelling extending above the right clavicle, which had gradually formed after a severe fit of coughing, and by an inequality of the pulsations of the two carotid arteries, that of the right being small and interrupted, that of the left full and more regular. The swelling was not painful except on deep pressure, and by that means one could, as it were, force back the elevated skin to the very base of the tumour, which, however, on remitting the pressure again, returned to its previous size and shape. The patient was occasionally seized with severe cough, and expectorated a good deal of bloody mucus; his skin was burning hot, his tongue dry and hard, his thirst insatiable.

Active antiphlogistic treatment was adopted, with some relief to the general symptoms; but by the end of the next day the emphysema, (for by it the swelling in the neck was produced,) had spread over the whole face and head and a part of the chest, and on the day after extended to the abdomen, and the arms and legs; the skin in some parts being so distended that it seemed ready to burst at every cough. In this condition the patient continued for some days, the cough and dyspnœa remaining very severe, though, by the steady use of calomel, purgatives, &c., the general health, and especially the very disordered state of the digestive organs, had been greatly remedied. On the seventh day from the beginning of the emphysema, several punctures were made in the skin of the chest, through which a large quantity of air escaped, but without producing any relief beyond reducing the tension of the skin; but, in spite of bandages, the first fit of coughing distended it again almost as much as ever. The author, therefore, determined to cure, if possible, the cough, and leave the emphysema to itself; and, succeeding in the former by narcotics, (belladonna, &c.) and tonics, the latter in a few days began gradually to decrease, and at length disappeared, leaving the patient however in a state of debility from which he only slowly recovered.

The author presumes that, in this case, emphysema of the upper part of the right lung was first produced, and that then some of its vesicles having burst, permitted the air to pass into some adhesions uniting its surface with that of the pleura, and thence into the cellular tissue of the whole body. And, rare as must be the coincidence of circumstances permitting such an event, we cannot suggest any more probable mode of explaining the phenomena of the case. Several cases similar to the present are recorded by authors.

IV. *Post-mortem Examination of a case of Ventro-Uterine Pregnancy, by J. Van Dam.*—This is a yet rarer case than the preceding. The patient was a woman who had twice borne children naturally. At the end of a third pregnancy labour came on, but after two days ceased without delivery, and the abdomen continued distended. Six days afterwards the motions of the child ceased, and from this time she suffered, during the seventeen subsequent months, repeated attacks of peritonitis. At the end of these the femur and some other bones of a putrid fœtus were discharged through the vagina, and the author determined to remove the remains by a Cæsarean operation. " By an incision through the abdominal walls," he says, " five inches long, and a hand's breadth to the left of the linea alba," (where the chief swelling was,) " a sac was exposed, whose walls were firm, half an inch thick, and adherent to those of the abdomen. A great quantity of pus and blood was let out of it, and the skeleton of a mature fœtus was removed still connected by ligaments, and fixed to the base of the sac so that it seemed to fill the whole cavity, and could with difficulty be removed. The sac itself appeared to extend down into the pelvis." After this she slowly recovered, but for several days fecal matter was discharged from the wound, proving that the sac was connected with the intestine. It gradually ceased, however, and the author supposed that the case had been one of utero-tubal pregnancy.

Seventeen months after her complete recovery, this woman became again pregnant, and continued well till in the last month of her term the motions of the fœtus suddenly ceased, and she became restless and dejected. A few days after this, the cicatrix of the former operation became elevated as if it would burst, and very tender, and it was determined to perform the Cæsarean section again; but on the morning of the day fixed she began suddenly to complain of tightness in the chest, convulsive motions ensued, her pulse sank, and death occurred in the course of three hours.

On examination the child was found within the peritoneum in an envelope occupying the epigastric and umbilical regions, surrounded by a transparent membrane, over which there was another firm fleshy covering traversed by numerous blood-vessels; this appeared to be the outermost membrane of the ovum unusually strong, and torn away from the border of the placenta which was in part visible at the right side. Below the child and sac was the uterus firmly adherent to the peritoneum and the cicatrix of the former operation: on their right lay a large portion of the placenta extending behind the right horn of the uterus. On exposing all these parts, the greater portion of the posterior wall of the uterus seemed to be deficient, its place being occupied by the membranous sac in which the child and its appendages were placed. The sac and the abdomen both contained a large quantity of blood.

" The posterior wall of the uterus, as well as the left superior angle of its anterior part, and the left Fallopian tube and ovary, were lost; the wall of the remaining portion was of a firm consistence, and nearly an inch thick; and it had attached to its border the strong vascular membranous sac which, as already described, lay loosely over the child, and which had formed the sac which had been ruptured before the woman's death. In the remaining portion of the cavity of the uterus there was still a portion of the placenta; the cervix was firm, very broad, and an inch and a half long: its canal was broad enough to admit a staff an inch wide and three lines thick." (p. 81.)

The author thinks (and probably with truth) that this cannot be regarded as, in the first instance, a case of completely extra-uterine fœtation, and for these reasons:

"Some of the bones of the fœtus were discharged through the vagina; the cavity from which in the operation the skeleton was removed, descended, funnel-like, into the pelvis: before the removal of the first fœtus, an instrument introduced into the uterus struck against a firm body, which was afterwards found to be the fœtal spine; after the operation pus as well as fæces passed through both the vagina and the external wound; and after death a sac was found containing all the products of conception, and communicating with, or rather, partially formed by, the still remaining portion of the uterus, whose cavity had also, without doubt, served for the reservoir of the former lengthened pregnancy.

"Hence it seems to follow," the author adds in explanation, "that the first pregnancy was completely intra-uterine; that this uterus, at the due time for delivery, underwent a partial weakening, and that the efforts of contraction produced, not delivery, but a *rupture,* the extent of which was not sufficient to permit the fœtus to pass wholly into the abdomen; the body remained in the uterus while the head passed probably into or through the wound. By the consequent inflammation . . . . . a covering was formed which filled the place of the destroyed part of the wall of the uterus, and which, after becoming adherent to the intestines, formed a communication with them by ulceration. The removal of the remains of the fœtus was followed by a contraction of the rest of the uterus. . . . . . Then a fresh pregnancy took place through the uninjured right ovary and Fallopian tube, and the development of the fœtus was completed in the uterus; but the distension of the walls produced a fresh rent. The efforts at delivery were fatal: the first contraction in the healthy part of the walls of the uterus soon overcame the resistance of the slightly organized part, filling up the lost wall, and produced the rupture which was followed by death." (p. 82.)

V. *On Congenital Closure of the Intestinal Canal and its Treatment, by J. J. F. La Cave.* In this paper there is detailed a good case of recovery from that defect after puncture of the anus and the use of bougies, and a rather large canula from time to time introduced into the rectum.

The account we have given of the contents of this volume is sufficient to prove that, though they be few and brief, the Transactions of the Medico-Chirurgical Society of Amsterdam have a merit not inferior to that of the publications of any medical society in Europe.

